# SeqMule: automated pipeline for analysis of human exome/genome sequencing data

**DOI:** 10.1038/srep14283

**Published:** 2015-09-18

**Authors:** Yunfei Guo, Xiaolei Ding, Yufeng Shen, Gholson J. Lyon, Kai Wang

**Affiliations:** 1 Zilkha Neurogenetic Institute, University of Southern California, Los Angeles, CA 90033, USA; 2 Department of Preventive Medicine, Keck School of Medicine, University of Southern California, Los Angeles, CA 90032, USA; 3 School of Forestry and Environment, Nanjing Forestry University, Nanjing, Jiangsu 210037, China; 4 Departments of Systems Biology and Biomedical Informatics, Columbia University, New York, NY 10032, USA; 5 Stanley Institute for Cognitive Genomics, Cold Spring Harbor Laboratory, New York, NY 11797, USA; 6 Utah Foundation for Biomedical Research, 150 S 100 W, Provo, UT, 84601, USA; 7 Department of Psychiatry & Behavioral Sciences, Keck School of Medicine, University of Southern California, Los Angeles, CA 90033, USA

## Abstract

Next-generation sequencing (NGS) technology has greatly helped us identify disease-contributory variants for Mendelian diseases. However, users are often faced with issues such as software compatibility, complicated configuration, and no access to high-performance computing facility. Discrepancies exist among aligners and variant callers. We developed a computational pipeline, SeqMule, to perform automated variant calling from NGS data on human genomes and exomes. SeqMule integrates computational-cluster-free parallelization capability built on top of the variant callers, and facilitates normalization/intersection of variant calls to generate consensus set with high confidence. SeqMule integrates 5 alignment tools, 5 variant calling algorithms and accepts various combinations all by one-line command, therefore allowing highly flexible yet fully automated variant calling. In a modern machine (2 Intel Xeon X5650 CPUs, 48 GB memory), when fast turn-around is needed, SeqMule generates annotated VCF files in a day from a 30X whole-genome sequencing data set; when more accurate calling is needed, SeqMule generates consensus call set that improves over single callers, as measured by both Mendelian error rate and consistency. SeqMule supports Sun Grid Engine for parallel processing, offers turn-key solution for deployment on Amazon Web Services, allows quality check, Mendelian error check, consistency evaluation, HTML-based reports. SeqMule is available at http://seqmule.openbioinformatics.org.

The development of next-generation sequencing (NGS) technologies has dramatically changed the landscape of human genetics research^1^,^2^,^3^,^4^,^5^,^6^. Identifying disease-contributory variants for various human genetic diseases will greatly improve diagnosis and facilitate development of therapies.

However, besides discrepancies associated with sequencing platforms^7^, there is still considerable variation across variant calling algorithms; for example, we previously reported SNV concordance of only 57.4% for 5 bioinformatics pipelines (SOAP, BWA-GATK, BWA-SNVer, GNUMAP, BWA-SAMtools), while 0.5–5.1% variants were called as unique to each pipeline^8^. Performance of aligners also varies under different sequencing error rates and indel distribution^9^. Yet few published pipelines offer two or more alternative aligner and variant calling programs^10^,^11^,^12^,^13^,^14^. While some workflow management systems do provide more flexibility^10^,^11^,^12^,^13^, local installation and configuration is highly challenging for average users. Therefore, there is a strong community need for a comprehensive and flexible pipeline that allows easy execution and integration of multiple tools.

There are multiple challenges for building such a pipeline. Installation and configuration poses the first problem, and the severity of this problem is evidenced by numerous attempts to address it^15^,^16^,^17^. Software libraries such as Bioconductor^15^ and Bioperl^16^, and web-based interfaces [e.g.^17^] all aim to provide ease of access. The diversity of bioinformatics tools has paradoxically given rise to one more layer of complexity. In a typical variant calling analysis, 4 to 6 tools might be required to perform QC (quality check), alignment, sorting, and variant calling. Ideally, the output from one program can be fed into another one as is. In real-world scenarios, this might not be the case. For instance, GATK does not accept output from SOAP2 aligner. Another issue is that constant and asynchronous development of the software would, from time to time, lead to loss of compatibility and break down of what was working. Even if compatibility issues can be solved, reproducibility will be difficult to maintain across highly heterogeneous pipelines. A pre-packaged virtual machine (VM) provides users with an alternative to address this problem^18^,^19^,^20^. However, having two operating systems running on the same machine means at least 1 CPU core and a few gigabytes of memory must be reserved for the host OS, and unavoidably limits the computational resources available for the guest system. Adding another layer of operating system also increases computational overhead by 13% to 28% compared with performance on a native system^19^. Finally, VM implementation reduces flexibility of software tools as a bundle and becomes difficult to deploy for average users without informatics skills.

To address the discrepancy issues without compromising ease of use, performance and reproducibility, we developed a computational pipeline, SeqMule, which performs a series of automated steps for identifying variants from NGS data. It integrates 5 alignment tools, 5 variant calling algorithms, and allows various combinations of them via modifying a text-based, human-readable configuration file. The intersection of sets of variants from different combinations of tools can be extracted to achieve higher accuracy, both in terms of sensitivity and specificity. Most setup procedure and analyses can be done with one-line commands. SeqMule also provides cluster-free parallel capability built on top of the variant callers, which could drastically reduce the time for variant calling by about an approximately linear factor of *N* (*N* is number of CPU cores). As far as we know, only GATK Queue and FreeBayes provide such parallelism among variant callers, but users have to manually set up a Queue or generate a region file for parallel processing. At the end of analysis, an HTML-based report will be prepared to show an overview for every step of the analysis, which helps assure users of data quality and appropriate analysis settings. We believe that SeqMule will be useful to easily and efficiently obtain variant calls from NGS data, and improve variant calling consistency and accuracy.

## Material and Methods

### Workflow

Currently, SeqMule integrates 5 popular mapping tools: BWA (including BWA-backtrack and BWA-MEM), Bowtie, Bowtie2, SOAP2, SNAP^21^,^22^,^23^,^24^,^25^, 5 variant calling algorithms: GATK (including GATKLite and version 3), SAMtools, VarScan 2, Freebayes, SOAPsnp^26^,^27^,^28^,^29^ and some accessory programs: FastQC, Picard, tabix and VCFtools^30^. Tools were selected based on their popularity, ease of use and performance. Of note, SNAP can be orders of magnitude faster compared with the popular aligner BWA-MEM^25^,^31^. All tools and related packages, except for those without open-source license, can be downloaded and installed by one single command with no need for root access. We actively maintain a list of programs and their related source code, database files to make sure there is no compatibility conflict under default settings.

**Figure 1:**
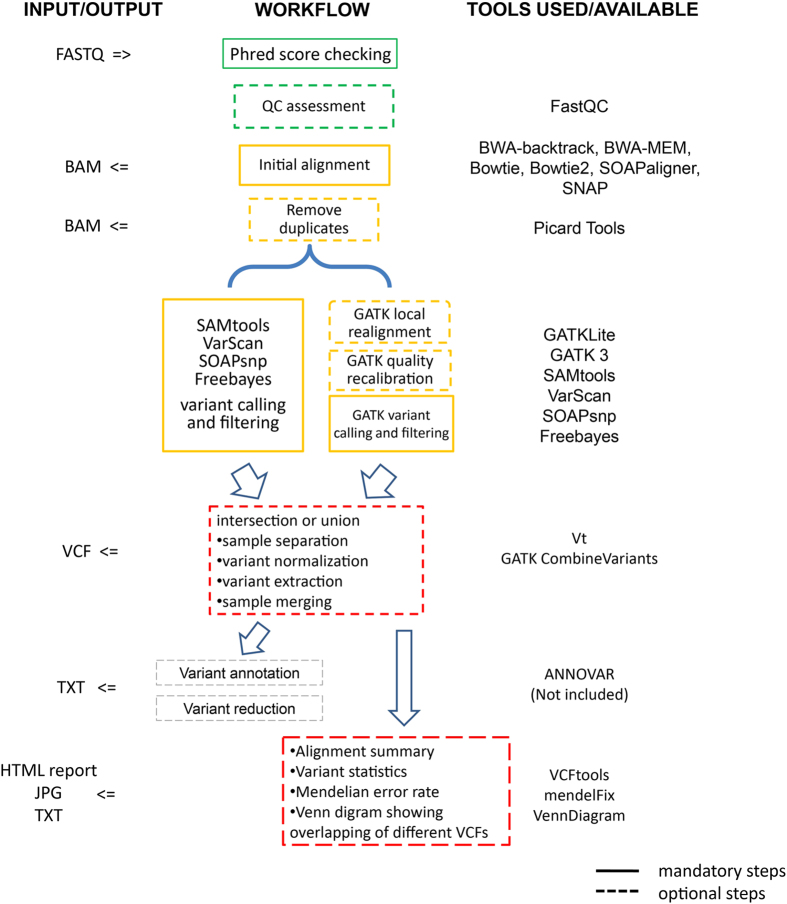
Scheme of SeqMule workflow and currently available tools in each step. Dashed line marks non-mandatory steps, solid line marks mandatory steps.

A workflow scheme is shown in Fig. 1. SeqMule takes FASTQ, gzipped FASTQ or BAM as input. Quality scores in FASTQ can be encoded either in Phred+33 scheme or in Phred+64 scheme. For FASTQ, SeqMule can automatically decide which scheme is used by examining the beginning of input. Other necessary files for analysis, including reference genomes, alignment indexes, known variant databases, can be downloaded via one-line built-in command from SeqMule website. In a typical pipeline, input data goes through QC, alignment, sorting, indexing, PCR duplicate removal, variant calling and report generation. All steps use default parameters. Reads with mapping quality larger than 30 (20 for SNAP) will be used for variant calling by default. Variants will be filtered following either recommended best practice (for GATK) or by depth threshold of 10 unless otherwise stated.

Multi-sample variant calling can be used if multiple sets of input data from the same lineage are supplied. BAM and VCF files are generated in the end. The VCF files are ready for downstream annotation and filtering analysis, which means users can feed them either to locally installed ANNOVAR program^32^ or to wANNOVAR web server^17^.

To allow various combinations of aligners and variant callers, SeqMule uses a specifically designed configuration file. The configuration file consists of key, value pairs in the form of ‘key=value’. Keys are categorized as global options, programs and local options, each with different prefixes. The prefixes for program keys also determine whether this program is mandatory and exclusive at a particular step. Programs can be either enabled or disabled by assigning 1 or 0 to the value of the corresponding keys. All settings are written in plain English with embedded help documentation alongside. Over 40 different combinations of aligners and variant callers have been tested and their configurations are readily available in ‘misc/predefined_config’ directory under SeqMule installation path. More combinations are left for the users to explore.

### Built-in parallel processing

**Figure 2:**
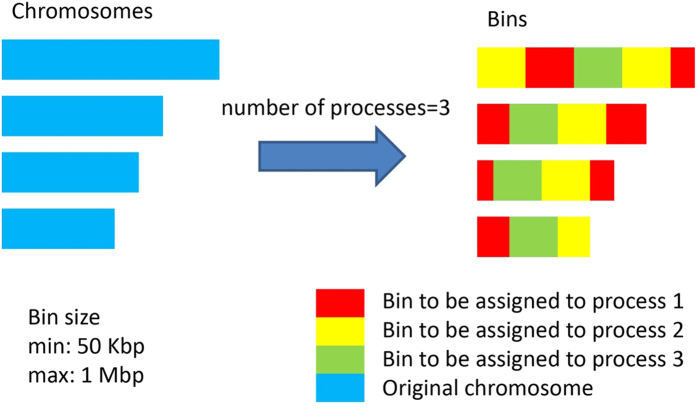
Bin generation and assignment. Assume we want to run our analysis using 3 processes. We need to split the chromosomes (blue) into 3 sets of bins. Bin size will be dynamically determined according to the total size of all chromosomes or user-defined regions. There is a minimum and maximum bin size to minimize the effects of a read spanning two bins and uneven coverage. In practice, the size limits shown in the figure work well from a couple of genes up to the whole genome. Bins are generated by walking through all regions to be analyzed. Subsequently, bins are assigned to each process by rotations. In the end, each process is expected to deal with approximately same numbers of reads.

Analysis can be run in a parallel fashion either via native support by variant callers (e.g. multi-processing option inside GATK) or via SeqMule’s built-in multi-processing framework. As is shown in Fig. 2, when SeqMule’s built-in multi-processing is enabled, it splits the genome into multiple equally sized bins, writes those bins into *N* BED files (*N* for number of CPU cores), and launches multiple processes to call variants over each region.

Each bin will be large enough to minimize the overhead costs of small bins, and small enough to have fine-grained genomic intervals. Currently the minimum bin size is set to be 50 Kbp while the maximum is 1 Mbp. All bins are assigned to each process by rotation (Fig. 2) so that two adjacent bins will not be analyzed by the same process. This assignment-by-rotation strategy is designed to avoid having too many reads processed by one thread due to uneven coverage^33^, therefore, all processes are expected to finish in similar amount of time. We compared variant calling time consumption under different max bin sizes using sample NA12878 from 1000 Genomes Project. Variants were called by SAMtools with 12 concurrent processes. Table S1 shows that the standard deviation increases as the max bin size grows from 500 Kbp to 20 Mbp. However, the maximum running time of child processes, or simply put, the overall variant calling time, does not necessarily increase as the max bin size becomes larger. Variant calling is fastest (194.9 minutes) when max bin size is 1 Mbp. All processes will be spawned by fork on a single machine, and so there is no need for cluster infrastructure. Different processes communicate with each other through a script file recording the status and command of each step. An execution manager (the parent process) is responsible for monitoring, starting and stopping all processes. Because some analyses may take hours or days to finish, the execution manager is designed to be able to stop and then resume the pipeline at any step. This feature comes in handy when users have to adjust some parameters (e.g. memory limit) after SeqMule aborts due to errors.

### Support for cluster environment

SeqMule supports analyzing large number of samples via Sun Grid Engine (SGE), a popular job scheduling system in cluster environment. Internally, when SeqMule generates tasks for each step for every sample, it also specifies their dependencies. The relationship results in a task dependency graph (TDG) with tasks as nodes and task interactions as edges. SeqMule then uses topological sorting to rearrange these tasks in linear fashion such that if task A is dependent on output from task B, task A will be put behind task B. Upon running, SeqMule runs tasks one by one and will not start a task unless all its dependent tasks are finished. With this design in place, many samples can be processed in parallel due to their nature of independence. When SGE is available, SeqMule submits each task to SGE and waits for it to finish.

### Consensus result generation

For SNVs, variant calls with the same chromosome and position fields are considered overlapping. Only such variants will be combined in consensus records. To combine two overlapping SNVs, alternative alleles in each record will be put together while the chromosome, position and reference columns remain unchanged.

For non-SNV variants, primarily consisting of indels, records with same chromosome, position and reference allele fields are considered overlapping. After combining, alternative alleles from all records will be put together. Same indels might be presented in different ways by different algorithms. For example, both ‘TGGG TGG‘ and ‘TG T’ can denote deletion of a G allele, but it is hard to tell which G is deleted in the first case^8^. In this circumstance, we apply ‘variant normalization’ in Vt (https://github.com/atks/vt) to first normalize all VCF files to the same standard before combining them. During normalization, all alleles will be left (5′) and right (3′) trimmed to remove superfluous nucleotides and be moved to the leftmost positions. Nevertheless, it is expected that concordance rate between different tools for indels are less than that for SNVs^8^.

When multiple samples are present in the same VCF file, it will be split into single-sample VCF before merging. After all VCFs from the same sample are merged, the resulting VCFs will be combined into one multi-sample VCF. Variant quality and genotype quality from the first input file will appear in the combined VCF. The files to be merged will be sorted based on the following priority: GATK>SAMtools>FreeBayes>VarScan>SOAPsnp. We assign variants ‘PASS’ in the filter field only if they are unfiltered in any one of the input VCF files. Consensus calls can be generated in different ways. Users are able to specify the minimum number of files in which each consensus call appears. For example, "2-out-of-4" consensus calls represent the calls found in at least 2 out of 4 input files.

### Analysis summary

When an analysis starts or finishes, SeqMule automatically examines the FASTQ, BAM, VCF and generates an HTML-based report showing various statistics. Important statistics include, but are not limited to, coverage curve, average coverage, coverage over capture region, percentage of capture region covered by at least N reads, Ti/Tv ratio, Heterozygote/Homozygote ratio. FASTQ statistics are calculated by FastQC; alignment statistics come from SAMtools; coverage statistics are done by SAMtools and SeqMule’s built-in program; VCFtools output most of the variant statistics.

Besides automatic generation of summary statistics, users can manually feed SeqMule with a multi-sample VCF from a family trio and obtain Mendelian error rates. The VCF file will first be converted to MAP and PED format used in PLINK^34^ to record all genotypes in a more compact fashion, Subsequently pedigree information, based on user specification, is added to the PED file. At last, SeqMule iterates through all variants in the offspring and counts number of calls with 0, 1, 2 IBS (identical by state) alleles from father or mother, number of allele drop-in events, number of allele drop-out events. The Mendelian error rate calculation is implemented with VCFtools, mendelFix^35^ along with custom Perl code.

In accordance with its multi-caller feature, SeqMule also generates a Venn Diagram for users to inspect overlapping of variant sets from different callers. Variants are split into SNVs and non-SNVs. Chromosome, position, reference allele and alternative allele of SNVs are used to determine whether two variants overlap. For non-SNVs, besides variant-normalization, records will first be intervalized by ignoring reference alleles and alternative alleles, and then extended 10 bp towards both ends. The extended intervals for each non-SNV are used to determine whether two variants overlap. This feature is implemented with VennDigram package^36^ and custom Perl code.

## Results

### Consistency and accuracy evaluation

**Figure 3:**
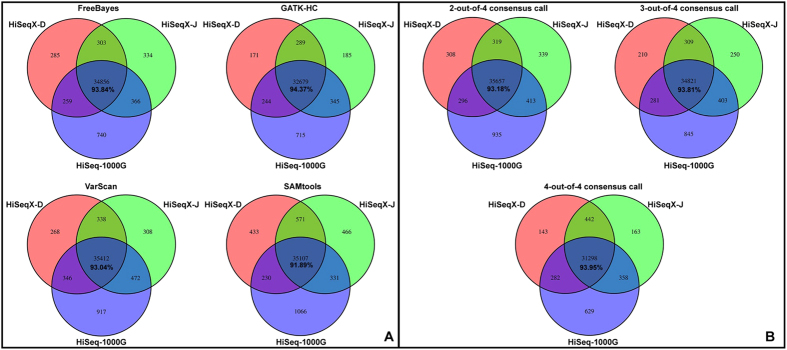
Consistency evaluation using 3 sequencing data sets for NA12878. The 3 data sets were generated by 1000 Genomes project and AllSeq (HiSeq-D, HiSeq-J). We used the same alignment algorithms (BWA-MEM) as the primary focus is to compare variant callers. For each data set, 4 variant callers were used to call variants (**A**), then the results from 4 callers were merged in different ways to get consensus calls (**B**). For each variant calling method, we plotted a Venn Diagram comparing overlapping of variants among the 3 data sets. The percentages show the proportion of variants shared by 3 data sets.

To demonstrate the consistency of calls on identical samples, we analyzed 3 data sets for NA12878 obtained from the 1000 Genomes project^37^ (sequenced by HiSeq and hereafter referred to as HiSeq-1000G) and from AllSeq (sequenced by HiSeq X Ten and hereafter referred to as HiSeqX-D, HiSeqX-J). For each data set, 4 variant callers, FreeBayes, SAMtools, VarScan and GATK HaplotypeCaller (GATK-HC) were used to call variants, then the results from 4 callers were merged in different ways to obtain consensus calls. For each variant calling method, we plotted a Venn diagram comparing the overlapping of variants among the 3 data sets (Fig. 3). The concordance rates range from 91.89% ~ 94.37% among all variant callers and consensus calls while GATK HaplotypeCaller calls give the highest concordance (94.37%).

**Figure 4:**
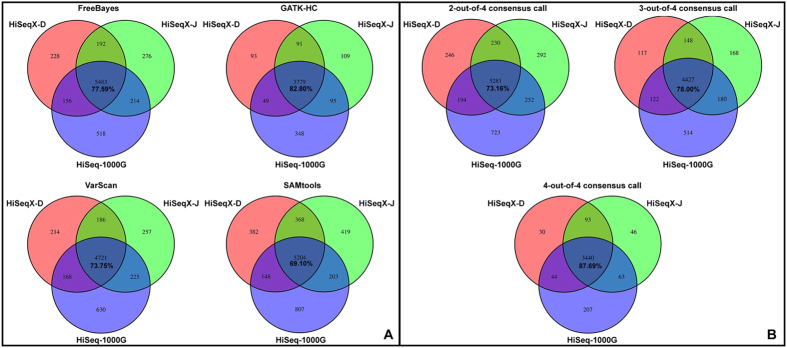
Consistency evaluation using rare variants from 3 sequencing data sets for NA12878. The 3 data sets were generated by 1000 Genomes project and AllSeq (HiSeq-D, HiSeq-J). We used the same alignment algorithms (BWA-MEM) as the primary focus is to compare variant callers. For each data set, 4 variant callers were used to call variants, variants with MAF > 1% were dropped. The remaining variants from 4 callers were merged in different ways to get consensus calls. For each variant calling method, we plotted a Venn Diagram comparing overlapping of variants among the 3 data sets (**A,B**). The percentages show the proportion of variants shared by 3 data sets.

As common variants are usually easy to detect, we later used ANNOVAR to filter the variants by minor allele frequency (MAF). Variants with MAF > 1% (1000 genomes project, October, 2014 release, European population) were discarded. The results (Fig. 4) show that for rare and novel variants, consistency rates are considerably lower, ranging from 69.10% ~ 87.69%. It suggests that most of the inconsistency in variant calling can be attributed to rare and novel variants. 4-out-of-4 consensus calls show the highest concordance (87.69%) under this scenario.

In addition to consistency, we also examined the precision, sensitivity and specificity. The 1000 genomes data set for NA12878 (HiSeq-1000G) was used in this case. All consensus calls and variants called by individual callers were compared with the gold standard from Genome In a Bottle (GIB) project and an Illumina HumanOmni2.5-8v1 SNP array (Figure S1-S2). For the comparison with GIB gold standard, precision rates range from 97.1% to 99.5% with GATK-HC being the highest; sensitivity rates range from 97.1% to 98.4%, with GATK-HC being the highest; specificity rates are all close to 100%. However, as the GIB gold standard was generated by GATK-HC, there is no doubt that the result is biased in favor of GATK. For the comparison with SNP array, precision rates range from 99.6% to 99.8% with 4-out-of-4 consensus calls being the highest; sensitivity rates range from 96.7% to 97.4% with 2-out-of-4 consensus call and GATK-HC both being the highest; specificity rates are all above 99.5%. These numbers show that all individual callers and consensus calls can give us accurate results in the regions covered by GIB gold standard and SNP array, indicating that the room for improvement may be somewhat limited.

### Aligner consistency evaluation

**Figure 5:**
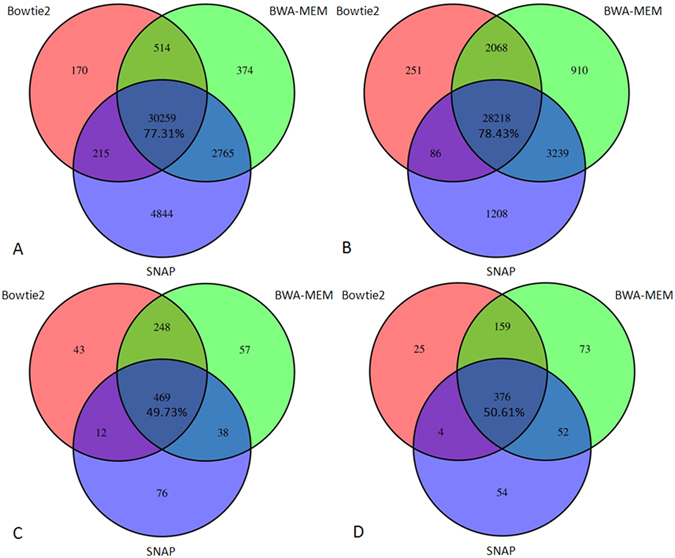
Variant concordance for different aligners. An exome data set was used to do alignment and call variants. For each variant caller, we used 3 aligners to map the reads. Two panels are SNVs, and two panels are non-SNVs (mostly indels). Panel A shows **SNV**s from SAMtools, panel B for **SNV**s from FreeBayes, panel C for **non-SNV**s from SAMtools, panel D for **non-SNV**s from FreeBayes.

To our knowledge, there is little comparison in literature regarding different aligners in the context of variant calling. It has been reported that there is differential performance for aligners when sequencing error rates and indel frequencies and sizes are varying^9^, so it is sensible to expect discrepancies associated with variant calling for different aligners. We used 2 variant callers, SAMtools and FreeBayes, and 3 aligners, BWA-MEM, Bowtie2 and SNAP, for each caller to evaluate concordance of variant calling on an exome data set^38^. Results in Fig. 5 show that concordance rates for SNVs are 77.31% for SAMtools, 78.43% for FreeBayes, respectively; concordance rates for non-SNVs (after normalization and extension) are 49.73% for SAMtools, 50.61% for FreeBayes, respectively. These results suggest that aligners may account for a considerable amount of difference in variant calling.

### Mendelian error evaluation

As family-based analysis is critical in Mendelian disease studies, we went on to examine Mendelian error rate by different methods in a previously published exome sequencing study on a family trio^38^. Two types of errors were counted: allele drop-in and allele drop-out. Allele drop-in (ADI) means that an offspring presents an allele that does not appear in either parent. Allele drop-out (ADO) means that an offspring misses an allele that should have been inherited from the parents. Sum of ADI and ADO is the total number of Mendelian errors. Mendelian error rate is the ratio of all Mendelian errors to all variant calls shared by the family trio (i.e. not marked as missing in any family member).

**Figure 6:**
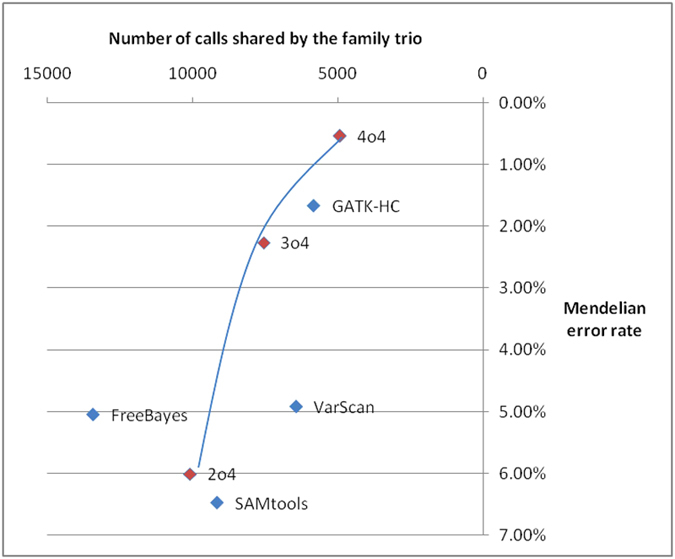
Mendelian error rate comparison for variant calling methods (MAF < 1%). 2o4 denotes calls from 2-out-of-4 consensus call set, 3o4 for 3-out-of-4 and 4o4 for 4-out-of-4. Only loci present in all family members are considered. ADI (allele drop in) and ADO (allele drop out) are counted as Mendelian errors. A trend line is added for consensus results. Only variants with MAF < 1% are shown here.

The result (Table S2) shows that individual variant callers generally returns 41.1 to 52.6 thousand trio calls with error rates between 1.78% and 2.88%. Among them, FreeBayes gives us the most trio calls (52.6 K) and GATK-HC gives us the lowest error rate (1.78%). For a single variant caller, it is hard to predict whether error rate is higher if the overall number of trio calls is small. In contrast, when we have fewer consensus trio calls, the Mendelian error rate is also lower. The lowest Mendelian error rate we can get is 0.66%, from 4-out-of-4 consensus calls for this data set, with 35.7 thousand trio calls. As most of our interests lie in rare and novel variants, we did variants filtering based on MAF. Variants with MAF >1% (1000 genomes project, October, 2014 release, European population) were discarded. The results show that generally Mendelian error rates become larger for rare and novel variants, ranging from 0.53% to 6.48% (Table S3, Fig. 6). Again, the lowest error rate was achieved by 4-out-of-4 consensus calls (0.53%), with nearly five thousand trio calls. Compared with results from individual callers, the consensus-call approach has more stable performance. It is also more flexible in that users can reduce Mendelian error rate when needed. In trio-based sequencing studies, researchers may want to examine the most reliable set of calls first to find low-hanging fruits, before delving deep into noisier call sets to find additional candidates.

### Performance evaluation

To evaluate the computational resources consumed by SeqMule, we used a whole genome sequencing data set (mentioned before as HiSeqX-D) with 818 million 151-bp paired end reads (30X coverage) for benchmarking. BWA-MEM or SNAP was used as the aligner. PicardTools was used to remove PCR duplicates. GATK HaploTypeCaller or FreeBayes was used to call variants. We used a machine equipped with 12-core Intel Xeon 2.66 GHz CPU and 48 Gigabytes of memory.

Table 1 details the computation time of SeqMule under different pipeline configurations. As is shown, conventional BWA-MEM and GATK-HC combination takes over 50 hours to analyze a whole genome, whereas SNAP+FreeBayes combination needs 36.3 hours. With quick mode (SeqMule’s built-in parallel framework) enabled, variant calling can run up to 12 times faster (12 CPU cores). However, this comes with the tradeoff of increased memory usage. For example, when speed is of primary concern, users can use the SNAP+FreeBayes combination. This combination requires less than 21 hours of running time and 31.3 Gigabytes of memory with quick mode enabled.

For users who are interested in analyzing exome data sets, we also did a similar comparison using an exome data set (138.8 million 90 bp-long paired-end reads, 113X coverage in target region). BWA-MEM was used as the aligner. PicardTools was used to remove PCR duplicates. GATK-HC was used to call variants. As is shown in Table S4, using more CPUs or processes reduces the running time in general. With quick mode enabled, variant calling can run 2 to 7 times faster depending on number of CPU cores used. On a machine with same configuration, the shortest running time is 317 minutes for the BWA-MEM + GATK-HC combination, and 264 minutes for the SNAP+FreeBayes combination (not shown in Table S4).

In the exome analysis, the running time for GATK-HC does not seem to vary with number of CPU cores when quick mode is disabled. This is perhaps due to the fact that GATK’s HaplotypeCaller only supports parallel execution with “-nct” option (enable multiple CPU threads per data thread) which does not scale well and has a constant overhead. The configuration files used are “snap_freebayes.config”, “bwa_gatk_HaplotypeCaller_norealn_norecal.config”. They can be found in seqmule/misc/predefined_config/. The software used includes FastQC (v0.11.2), Picard (v1.115), SAMtools (v0.1.19–44428cd), FreeBayes (v0.9.14-14-gb00b735), SNAP (v1.0beta.16), BWA-MEM (v0.7.10-r789), GATK (v3.1-1-g07a4bf8).

### Mendelian disease disease-contributory variant identification

We used exome sequencing data from a previously reported family trio^38^ to demonstrate the applicability and ease of use of SeqMule. The proband is a 28-year-old Caucasian male diagnosed with idiopathic hemolytic anemia. This phenotype was not observed either in his parents nor his siblings. With Agilent SureSelect Human All Exon kit and Illumina HiSeq 2000, we obtained 97 million paired-end reads per sample with designed capture regions covered at 82x on average. Initial alignment was done by BWA-MEM algorithm, followed by Picard removal of duplicates. GATK HaplotypeCaller, SAMtools, VarScan were then used to call variants. Multi-sample variant calling was enabled, so all 3 samples were supplied to each of the variant callers. We filtered results based on criteria recommended by each algorithm. Subsequently we extracted consensus variants from 3 sets of filtered variants (in 3-out-of-3 fashion), and reduced number of variants from 34 thousand per caller to 31 thousand on average. These steps were performed by executing one single SeqMule command given that the combination of aligner and variant callers has been tested before.

Next we utilized the ANNOVAR “variants reduction” pipeline^32^, under a recessive disease model. Synonymous, non-splicing variants and variants observed in the 1000 Genomes Projects and NHLBI-ESP 6500 exome project with minor allele frequency (MAF) >1% in European populations^39^ were filtered out. The filtering was done with one single ANNOVAR command for each subject (variants_reduction.pl -protocol nonsyn_splicing,1000g2012apr_all,esp6500_ea,recessive -operation g,f,f,m -aaf_threshold 0.01 input.father.avinput annovar/humandb/ -buildver hg19).

In the remaining variants, father and son shared 236 variants, while mother and son shared 253. There are 64 genes overlapping between the two sets of shared variants, 32 of which are homozygous and 6 are compound heterozygous in the proband. Manual examination of candidate genes easily identified mutations in *PKLR* that likely contribute to the disease. *PKLR* harbours two heterozygous variants that are predicted by multiple scores to be deleterious, and has been confirmed by us as the disease-contributory gene in biochemical tests^38^. This example demonstrated that SeqMule can reveal disease-contributory variants easily with minimal efforts from the users, therefore greatly facilitating genetic studies on human genetic diseases.

## Discussion

In summary, SeqMule is a comprehensive, user-friendly, flexible and efficient tool for analyzing human exome and genome sequencing data. Five alignment tools, five variant callers and various accessory programs are included to provide users with numerous choices.

Different call sets can be integrated to produce more reliable results in terms of consistency and Mendelian error rate. However, if users are solely interested in rare variants and *de novo* point mutations from family-based samples, they will have more gain in power and Mendelian consistency by using more specialized tools such as PolyMutt^40^, DenovoGear^41^ and FamSeq^42^. Test results on our own data suggest that PolyMutt can dramatically reduce Mendelian errors (0.2% for FreeBayes, 0.0% for GATK-HC, 0.3% for SAMtools, data same as in Fig. 6) by utilizing a family-aware calling framework. When sample size is large, variant quality can also be improved by imputation-based methods^43^.

SeqMule’s built-in parallel processing capability can improve current variant calling by a number of times without involving high-performance computing infrastructure or complicated configuration. Besides all of these features, SeqMule uses one-line commands for complicated tasks wherever possible, making it extremely easy to download, install, configure and run a large number of bioinformatics tools.

Popular bioinformatics platforms, such as Galaxy, make a number of bioinformatics tools very accessible. Users just upload their data and start analysis right away. But the restricted storage, limited data transfer speed and prolonged job queuing time makes it impractical to use when users have non-trivial amount of data.

The option of launching a Galaxy instance^44^ or other pipeline in the cloud is likely to overcome some of the obstacles mentioned above without cumbersome local installation. But, as a free, public resource open to all biologists, such a platform is usually built towards serving a large population who are not only interested in variant discovery, but also many other types of analyses. In contrast, SeqMule is tailored for exome or genome sequence analysis in the context of human genetic disease study and therefore hosts many features optimized for this purpose, such as consensus call generation, multi-sample variant calling, Mendelian error rate statistics, and HTML-based summary, among other things. Besides, SeqMule is more agile because new tools, once they are integrated into SeqMule by developers, can be added with just one update command (see SeqMule’s manual).

In addition to platform solutions, there are quite a few standalone pipelines for variant analysis, such as HugeSeq^45^, ngs_backbone^46^ and bcbio-nextgen (https://github.com/chapmanb/bcbio-nextgen). HugeSeq integrates one aligner and two SNP/indel callers (SAMtools and GATK), and was last updated one year ago. ngs_backbone integrates one aligner (BWA) and one variant caller (GATK). bcbio-nextgen has two aligners (BWA, NovoAlign) and four variant callers (GATK, FreeBayes, Platypus, SAMtools). Since NovoAlign is a proprietary aligner not free to all researchers, none of these pipelines provides alternative open-source mappers. In view of discrepancies associated with aligners^9^ (Fig. 5), it is important to offer users multiple open-source alignment programs.

Many users have used SeqMule to analyze their sequencing data and obtained meaningful results^47^,^48^,^49^. With the rapid development and deployment of next-generation sequencing technologies, we expect that SeqMule will facilitate analysis of the upcoming massive amounts of sequencing data to expedite discoveries for human genetic diseases.

## Additional Information

**How to cite this article**: Guo, Y. *et al.* SeqMule: automated pipeline for analysis of human exome/genome sequencing data. *Sci. Rep.* **5**, 14283; doi: 10.1038/srep14283 (2015).

## Supplementary Material

Supplementary Information

## Figures and Tables

**Table 1:** Time consumption under different configurations.

Quick Mode	Aligner	Variant caller	CPU (number of cores)	Max Memory Used (G)	Alignment Time (hour)	Variant Calling Time (hour)	Total Time (hour)
No	BWA	GATK-HC	12	19.8	13.5	22.6	54.0
Yes	BWA	GATK-HC	12	43.2	13.2	19.8	51.1
No	SNAP	FreeBayes	12	31.3	5.7	16.0	36.3
Yes	SNAP	FreeBayes	12	31.3	5.3	1.3	20.5

A human whole genome data set (818 million 151 bp-long paired-end reads, 30X coverage) was aligned with BWA-MEM and SNAP. PCR duplicates were removed by Picardtools. Variants were called by GATK HaplotypeCaller and FreeBayes. Quick mode here denotes SeqMule’s built-in parallel framework. Built-in parallel capability is always turned on for underlying 3^rd^ party algorithms (e.g. GATK’s -nt option).

## References

[b1] Rabbani B., Mahdieh N., Hosomichi K., Nakaoka H. & Inoue I. Next-generation sequencing: impact of exome sequencing in characterizing Mendelian disorders. J Hum Genet 57, 621–632 (2012). 22832387 10.1038/jhg.2012.91

[b2] Bamshad M. J. *et al.* Exome sequencing as a tool for Mendelian disease gene discovery. Nat Rev Genet 12, 745–755 (2011). 21946919 10.1038/nrg3031

[b3] Meyerson M., Gabriel S. & Getz G. Advances in understanding cancer genomes through second-generation sequencing. Nat Rev Genet 11, 685–696 (2010). 20847746 10.1038/nrg2841

[b4] Mardis E. R. The impact of next-generation sequencing technology on genetics. Trends in genetics: TIG 24, 133–141 (2008). 18262675 10.1016/j.tig.2007.12.007 PMC2680276

[b5] Morozova O. & Marra M. A. Applications of next-generation sequencing technologies in functional genomics. Genomics 92, 255–264 (2008). 18703132 10.1016/j.ygeno.2008.07.001

[b6] Genomes Project C. *et al.* A map of human genome variation from population-scale sequencing. Nature 467, 1061–1073 (2010). 20981092 10.1038/nature09534 PMC3042601

[b7] Lam H. Y. *et al.* Performance comparison of whole-genome sequencing platforms. Nat Biotechnol 30, 78–82 (2012). 22178993 10.1038/nbt.2065 PMC4076012

[b8] O'Rawe J. *et al.* Low concordance of multiple variant-calling pipelines: practical implications for exome and genome sequencing. Genome Med 5, 28 (2013). 23537139 10.1186/gm432 PMC3706896

[b9] Ruffalo M., LaFramboise T. & Koyuturk M. Comparative analysis of algorithms for next-generation sequencing read alignment. Bioinformatics 27, 2790–2796 (2011). 21856737 10.1093/bioinformatics/btr477

[b10] Hull D. *et al.* Taverna: a tool for building and running workflows of services. Nucleic Acids Res 34, W729–732 (2006). 16845108 10.1093/nar/gkl320 PMC1538887

[b11] Reich M. *et al.* GenePattern 2.0. Nat Genet 38, 500–501 (2006). 16642009 10.1038/ng0506-500

[b12] Abouelhoda M., Issa S. A. & Ghanem M. Tavaxy: integrating Taverna and Galaxy workflows with cloud computing support. BMC Bioinformatics 13, 77 (2012). 22559942 10.1186/1471-2105-13-77 PMC3583125

[b13] Goecks J., Nekrutenko A., Taylor J. & Galaxy, T. Galaxy: a comprehensive approach for supporting accessible, reproducible, and transparent computational research in the life sciences. Genome Biol 11, R86 (2010). 20738864 10.1186/gb-2010-11-8-r86 PMC2945788

[b14] Pabinger S. *et al.* A survey of tools for variant analysis of next-generation genome sequencing data. Brief Bioinform 15, 256–278 (2014). 23341494 10.1093/bib/bbs086 PMC3956068

[b15] Gentleman R. C. *et al.* Bioconductor: open software development for computational biology and bioinformatics. Genome Biol 5, R80 (2004). 15461798 10.1186/gb-2004-5-10-r80 PMC545600

[b16] Stajich J. E. *et al.* The Bioperl toolkit: Perl modules for the life sciences. Genome Res 12, 1611–1618 (2002). 12368254 10.1101/gr.361602 PMC187536

[b17] Chang X. & Wang K. wANNOVAR: annotating genetic variants for personal genomes via the web. Journal of medical genetics 49, 433–436 (2012). 22717648 10.1136/jmedgenet-2012-100918 PMC3556337

[b18] Krampis K. *et al.* Cloud BioLinux: pre-configured and on-demand bioinformatics computing for the genomics community. BMC Bioinformatics 13, 42 (2012). 22429538 10.1186/1471-2105-13-42 PMC3372431

[b19] Nocq J., Celton M., Gendron P., Lemieux S. & Wilhelm B. T. Harnessing virtual machines to simplify next-generation DNA sequencing analysis. Bioinformatics 29, 2075–2083 (2013). 23786767 10.1093/bioinformatics/btt352

[b20] Angiuoli S. V. *et al.* CloVR: a virtual machine for automated and portable sequence analysis from the desktop using cloud computing. BMC Bioinformatics 12, 356 (2011). 21878105 10.1186/1471-2105-12-356 PMC3228541

[b21] Li H. & Durbin R. Fast and accurate short read alignment with Burrows-Wheeler transform. Bioinformatics 25, 1754–1760 (2009). 19451168 10.1093/bioinformatics/btp324 PMC2705234

[b22] Langmead B., Trapnell C., Pop M. & Salzberg S. L. Ultrafast and memory-efficient alignment of short DNA sequences to the human genome. Genome Biol 10, R25 (2009). 19261174 10.1186/gb-2009-10-3-r25 PMC2690996

[b23] Langmead B. & Salzberg S. L. Fast gapped-read alignment with Bowtie 2. Nat Methods 9, 357–359 (2012). 22388286 10.1038/nmeth.1923 PMC3322381

[b24] Li R. *et al.* SOAP2: an improved ultrafast tool for short read alignment. Bioinformatics 25, 1966–1967 (2009). 19497933 10.1093/bioinformatics/btp336

[b25] Zaharia M. *et al.* Faster and More Accurate Sequence Alignment with SNAP. ArXiv e-prints 1111, 5572, 2011arXiv1111.5572Z (2011).

[b26] McKenna A. *et al.* The Genome Analysis Toolkit: a MapReduce framework for analyzing next-generation DNA sequencing data. Genome Res 20, 1297–1303 (2010). 20644199 10.1101/gr.107524.110 PMC2928508

[b27] Li H. *et al.* The Sequence Alignment/Map format and SAMtools. Bioinformatics 25, 2078–2079 (2009). 19505943 10.1093/bioinformatics/btp352 PMC2723002

[b28] Koboldt D. C. *et al.* VarScan 2: somatic mutation and copy number alteration discovery in cancer by exome sequencing. Genome Res 22, 568–576 (2012). 22300766 10.1101/gr.129684.111 PMC3290792

[b29] Li R. *et al.* SNP detection for massively parallel whole-genome resequencing. Genome Res 19, 1124–1132 (2009). 19420381 10.1101/gr.088013.108 PMC2694485

[b30] Danecek P. *et al.* The variant call format and VCFtools. Bioinformatics 27, 2156–2158 (2011). 21653522 10.1093/bioinformatics/btr330 PMC3137218

[b31] R. Pandya W. B. *et al.* SNAP: fast, accurate sequence alignment enabling biological applications. ASHG meeting 2014, San Diego (2014).

[b32] Wang K., Li M. & Hakonarson H. ANNOVAR: functional annotation of genetic variants from high-throughput sequencing data. Nucleic Acids Res 38, e164 (2010). 20601685 10.1093/nar/gkq603 PMC2938201

[b33] Wang W., Wei Z., Lam T. W. & Wang J. Next generation sequencing has lower sequence coverage and poorer SNP-detection capability in the regulatory regions. Scientific reports 1, 55 (2011). 22355574 10.1038/srep00055 PMC3216542

[b34] Purcell S. *et al.* PLINK: a tool set for whole-genome association and population-based linkage analyses. American journal of human genetics 81, 559–575 (2007). 17701901 10.1086/519795 PMC1950838

[b35] Utsunomiya Y. T. *et al.* mendelFix: a Perl script for checking Mendelian errors in high density SNP data of trio designs. arXiv:1306.2243 (2013).

[b36] Chen H. VennDiagram: Generate high-resolution Venn and Euler plots. CRAN (2011).

[b37] Altshuler D. M. *et al.* An integrated map of genetic variation from 1,092 human genomes. Nature 491, 56–65 (2012). 23128226 10.1038/nature11632 PMC3498066

[b38] Lyon G. J. *et al.* Exome sequencing and unrelated findings in the context of complex disease research: ethical and clinical implications. Discovery medicine 12, 41 (2011). 21794208 PMC3544941

[b39] Genomes Project C. *et al.* An integrated map of genetic variation from 1,092 human genomes. Nature 491, 56–65 (2012). 23128226 10.1038/nature11632 PMC3498066

[b40] Li B. *et al.* A likelihood-based framework for variant calling and de novo mutation detection in families. PLoS genetics 8, e1002944 (2012). 23055937 10.1371/journal.pgen.1002944 PMC3464213

[b41] Ramu A. *et al.* DeNovoGear: de novo indel and point mutation discovery and phasing. Nat Methods 10, 985–987 (2013). 23975140 10.1038/nmeth.2611 PMC4003501

[b42] Peng G. *et al.* Rare variant detection using family-based sequencing analysis. Proc Natl Acad Sci USA 110, 3985–3990 (2013). 23426633 10.1073/pnas.1222158110 PMC3593912

[b43] Nielsen R., Korneliussen T., Albrechtsen A., Li Y. & Wang J. SNP calling, genotype calling, and sample allele frequency estimation from New-Generation Sequencing data. PLoS One 7, e37558 (2012). 22911679 10.1371/journal.pone.0037558 PMC3404070

[b44] Afgan E. *et al.* Galaxy CloudMan: delivering cloud compute clusters. BMC Bioinformatics 11 Suppl 12, S4 (2010). 21210983 10.1186/1471-2105-11-S12-S4 PMC3040530

[b45] Lam H. Y. *et al.* Detecting and annotating genetic variations using the HugeSeq pipeline. Nat Biotechnol 30, 226–229 (2012). 22398614 10.1038/nbt.2134 PMC4720384

[b46] Blanca J. M., Pascual L., Ziarsolo P., Nuez F. & Canizares J. ngs_backbone: a pipeline for read cleaning, mapping and SNP calling using next generation sequence. BMC Genomics 12, 285 (2011). 21635747 10.1186/1471-2164-12-285 PMC3124440

[b47] Shi L. *et al.* Genotype-first inverted question mark approaches on a curious case of idiopathic progressive cognitive decline. BMC medical genomics 7, 66 (2014). 25466957 10.1186/s12920-014-0066-9 PMC4267425

[b48] Jia H., Guo Y., Zhao W. & Wang K. Long-range PCR in next-generation sequencing: comparison of six enzymes and evaluation on the MiSeq sequencer. Scientific reports 4, 5737 (2014). 25034901 10.1038/srep05737 PMC4102922

[b49] Zhang X. *et al.* Exome sequencing on malignant meningiomas identified mutations in neurofibromatosis type 2 (NF2) and meningioma 1 (MN1) genes. Discov Med 18, 301–311 (2014). 25549701 PMC4720499

