# Supplementary material for: SeqMule: automated pipeline for analysis of human exome/genome sequencing data
Source: Sci Rep. 2015 Sep 18;5:14283. doi: 10.1038/srep14283 (PMC4585643; doi:10.1038/srep14283)

SeqMule: automated pipeline for analysis of human exome/genome sequencing data

Yunfei Guo[1,2], Xiaolei Ding[3], Yufeng Shen[4], Gholson J. Lyon[5,6], Kai Wang[1,2,6,7,*]

[1]Zilkha Neurogenetic Institute, University of Southern California, Los Angeles, CA 90033, USA
[2]Department of Preventive Medicine, Keck School of Medicine, University of Southern California, Los Angeles, CA 90032, USA
[3]School of Forestry and Environment, Nanjing Forestry University, Nanjing, Jiangsu 210037, China
[4]Departments of Systems Biology and Biomedical Informatics, Columbia University, New York, NY 10032, USA
[5]Stanley Institute for Cognitive Genomics, Cold Spring Harbor Laboratory, New York, NY 11797, USA
[6]Utah Foundation for Biomedical Research, 150 S 100 W, Provo, UT, 84601, USA
[7]Department of Psychiatry & Behavioral Sciences, Keck School of Medicine, University of Southern California, Los Angeles, CA 90033, USA

* To whom correspondence should be addressed. Tel: +1 (323) 442-3917; Fax: +1 (323) 442-2145; Email: kaiwang@usc.edu

Supplementary information

Table S1. Variant calling time consumption and max bin size in quick mode. Sample NA12878 from 1000 Genomes Project was used to benchmark variant calling time with different max bin sizes in quick mode. Variants were called by SAMtools with 12 concurrent processes. K stands for thousand, M for million. Row minimums are highlighted by bold face. The statistics was calculated based on running time of all child processes. The result shows that the smaller the max bin size is, the smaller the standard deviation is, and that maximum running time does not increase linearly as max bin size grows. The shortest maximum running time is obtained when max bin size is 1 Mbp.

Max Bin Size (bp)	500K	1M	5M	10M	20M
Average running time (min)	209.2	**191.4**	191.5	210.0	209.6
Minimum running time (min)	207.7	188.0	**180.5**	189.6	188.6
Maximum running time (min)	211.4	**194.9**	199.4	233.1	253.0
Standard Deviation	**1.3**	1.8	5.4	12.3	16.5

Table S2. Mendelian error rate comparison for variant calling methods. Allele drop in (ADI) means that an offspring presents an allele that does not appear in either parent. Allele drop out (ADO) means that an offspring misses an allele that should have been inherited from the parents.

	Number of calls shared by the family trio	Number of allele drop in	Number of allele drop out	Total number of Mendelian errors	Proportion of Mendelian errors
GATK HaplotypeCaller	44559	215	579	794	1.78%
SAMtools	48586	769	631	1400	2.88%
FreeBayes	52609	797	321	1118	2.13%
VarScan	41102	360	542	902	2.19%
Consensus (2 out of 4)	49741	730	796	1526	3.07%
Consensus (3 out of 4)	45748	228	603	831	1.82%
Consensus (4 out of 4)	35690	6	230	236	0.66%

Table S3. Mendelian error rate comparison for variant calling methods (MAF<1%). Allele drop in (ADI) means that an offspring presents an allele that does not appear in either parent. Allele drop out (ADO) means that an offspring misses an allele that should have been inherited from the parents.

	Number of calls shared by the family trio	Number of allele drop in	Number of allele drop out	Total number of Mendelian errors	Proportion of Mendelian errors
GATK HaplotypeCaller	5831	46	51	97	1.66%
SAMtools	9155	471	122	593	6.48%
FreeBayes	13426	644	34	678	5.05%
VarScan	6428	228	88	316	4.92%
Consensus (2 out of 4)	10090	447	159	606	6.01%
Consensus (3 out of 4)	7553	84	87	171	2.26%
Consensus (4 out of 4)	4934	4	22	26	0.53%

Table S4. Time consumption under different configurations. A human exome data set (138.8 million 90bp-long paired-end reads, 113X coverage in target region) was aligned with BWA-MEM. PCR duplicates were removed by Picardtools. Variants were called by GATK HaplotypeCaller. Quick mode here denotes SeqMule's built-in parallel framework. Built-in parallel capability is always turned on for underlying 3rd party algorithms.

Quick Mode Enabled	CPU (number of cores)	Max Memory Used (G)	Variant Calling Time (min)	Time (min)	Time Saving Compared With Analysis Using 1 CPU
No	1	7.03	217.5	910	0.00%
No	2	8.17	208.4	695	23.63%
No	4	11.90	189.9	540	40.66%
No	8	16.27	203.6	529	41.87%
No	12	20.34	192.5	474	47.91%
Yes	2	8.17	117.8	606	33.41%
Yes	4	14.67	51.5	385	57.69%
Yes	8	28.98	40.3	346	61.98%
Yes	12	43.29	31.1	317	65.16%

Figure S1. Compare individual callers with Genome In a Bottle Gold Standard. Results from 4 individual variant callers, GATK HaplotypeCaller (gatk_hc), Freebayes, SAMtools and VarScan were uploaded to http://www.bioplanet.com/gcat to be compared against gold standard from Genome In a Bottle project (Version 2.18) and Illumina HumanOmni2.5-8v1 SNP array.

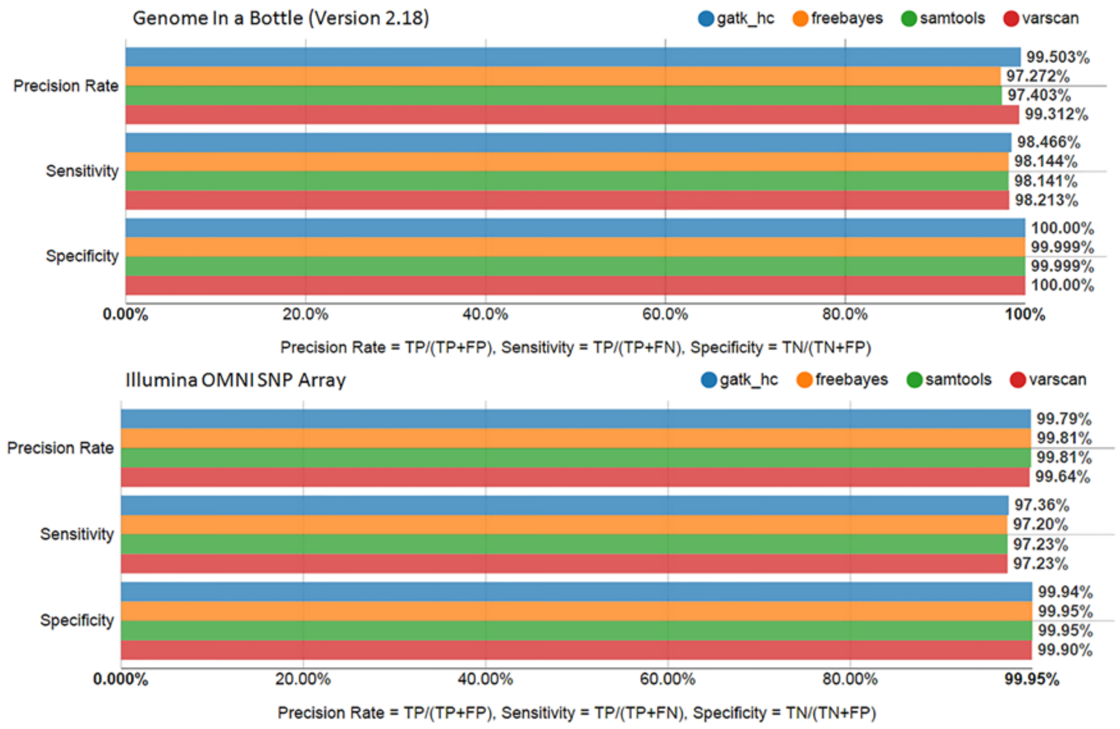

Figure S2. Compare consensus results with Genome In a Bottle Gold Standard. Consensus calls were generated by combining results from individual variant callers in different ways, namely 2-out-of-4, 3-out-of-4 and 4-out-of-4. Then they were uploaded to http://www.bioplanet.com/gcat to be compared against gold standard from Genome In a Bottle project (Version 2.18) and Illumina HumanOmni2.5-8v1 SNP array. Result from GATK HaplotypeCaller (GATK-HC) is also shown as a reference.

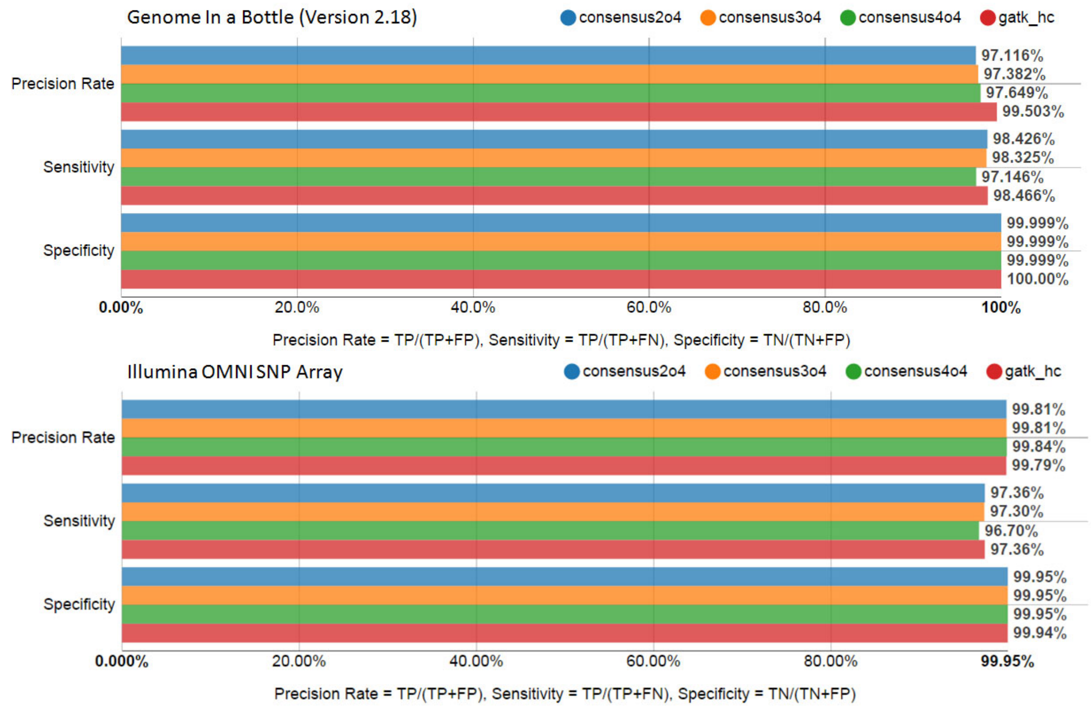

Supplement: Supplementary Information [file srep14283-s1.pdf]
